# Application of stereotactic biopsy for diagnosing intracranial lesions in patients with AIDS in China: Report of 7 cases

**DOI:** 10.1097/MD.0000000000005526

**Published:** 2016-12-09

**Authors:** Ji-bo Zhang, Kai Fu, Rui Gong, Xue-meng Liu, Li-dao Chen, Yong-xi Zhang, Gui-fang Yang, Jie Zhang

**Affiliations:** a Department of Neurosurgery; b Department of Infectious Disease; c Department of Pathology, Zhongnan Hospital of Wuhan University, Wuhan, China.

**Keywords:** AIDS, China, intracranial lesion, stereotactic biopsy

## Abstract

**Rationale:**

The aim of the study was to evaluate stereotactic biopsy for diagnosing intracranial lesions in patients with AIDS.

**Patient concerns:**

Seven AIDS patients with an intracranial lesion who underwent stereotactic biopsy were included in this retrospective study (4 males and 3 females, 15 to 49 years old). The patients’ disease history ranged from 1 month to 1 year. The samples were examined by hematoxylin-eosin (HE) staining and immunohistochemical examination.

**Diagnoses, interventions and outcomes:**

All patients were successfully sampled, and the histological results showed inflammation in 4 cases, toxoplasma gondii infection in 1 case, astrocytoma in 1 case, and abscess in 1 case. The clinical diagnosis included toxoplasma encephalitis (TE) in 2 cases, cryptococcus encephalitis in 2 cases, cytomegalovirus (CMV) encephalitis in 2 case, tubercular abscess in 1 case, astrocytoma in 1 case, and co-infection of TE with Cryptococcus infection in 1 patient. The clinical diagnosis was made according to the plasma and cerebrospinal fluid (CSF) laboratory testing, the imaging data and the histological findings. The diagnostic yield was 100%, and the post-operation morbidity was 14.3% (1/7) with an asymptomatic haemorrhage and seizure in 1 case. There was no operation-related mortality. Patients were followed up for 6 months to 6 years; 1 case fully recovered, 4 cases significantly improved in symptoms, and 2 died.

**Lessons:**

Stereotactic biopsy is a safe and effective way of diagnosing intracranial lesions in patient with AIDS. It is helpful for the differential diagnosis and for choosing a suitable therapy. Due to the broad spectrum of nervous system abnormalities in AIDS, histological findings are very valuable. However, histology is not a unique tool for making a definite diagnosis, whereas the combination of molecular pathology and stereotactic biopsy should play a more important role in the future.

## 1 Introduction

AIDS (acquired immune deficiency syndrome), a chronic systemic disease, is caused by HIV (human immunodeficiency virus). It remains a major global public health issue. The WHO (World Health Organization) report shows that 2.1 (1.8–2.4) million people are newly infected with HIV, and 1.1 (0.94–1.3) million people died from HIV-related causes globally in 2015. Approximately 36.7 (34.0–39.8) million people were living with HIV at the end of 2015.^[1]^

It is clear that the central nervous system (CNS) is a primary target for HIV.^[2]^ The immunodeficiency caused by the virus can lead to a plethora of other opportunistic cerebral infections as well as neoplasia.^[3]^ CNS involvement presents as the first clinical symptom of AIDS in ∼10% to 20% of patients.^[4]^ Many (40–60%) patients develop neurological abnormalities during the disease course.^[5]^ Moreover, 75% to 80% of all AIDS patients show neuropathological changes in the CNS upon brain autopsy,^[6]^ and multiple pathologies are found in 17% of cases.^[7]^

AIDS-related nervous system abnormalities can be classified into the following 5 types: HIV-related primary infections, such as aseptic meningitis and encephalitis; opportunistic infections of the nervous system, such as cytomegalovirus (CMV) encephalomyelitis, toxoplasma encephalitis (TE), progressive multifocal leukoencephalopathy (PML), herpes viral encephalitis, Cryptococcal meningitis, and Mycobacterium tuberculosis infection; neoplasms, commonly non-Hodgkin's lymphoma (NHL) and occasionally Kaposi's sarcoma; cerebrovascular diseases and peripheral neuropathy.^[4]^ With the rapid development and broad use of medical imaging technology, most AIDS-related brain lesions can be found at an early stage. However, they cannot provide an adequate diagnosis. Usually, a histopathological examination is still needed for choosing a suitable treatment. Brain biopsy is indicated in the absence of clinical and radiologic improvement after empiric therapy.^[5]^

Stereotactic biopsy has been considered with a high diagnostic yield and safety for diagnosing intracranial lesions.^[8,9]^ There are no previous reports on stereotactic biopsy performed in AIDS patients with intracranial lesions in China. In this study, 7 AIDS patients with intracranial lesions who underwent stereotactic biopsy were retrospectively analysed.

## 2 Patients and methods

### 2.1 General information and symptoms

From November 2010 to January 2016, 7 AIDS patients with intracranial lesions underwent stereotactic biopsy. There were 4 males and 3 females who were 15 to 49 (mean: 36) years old. The disease history ranged from 1 month to 1 year. Informed consent was collected in advance of biopsy from every patient and their family members. The clinical symptoms included headache, dizziness, limb weakness, fever and cough, dysphagia, vision loss, and epilepsy (Table 1). Preoperative computed tomography (CT) and magnetic resonance imaging (MRI) scanning were performed in all cases. After 3 weeks of empiric treatment, neither clinical symptoms nor imaging improvement were found.

**Table 1:**
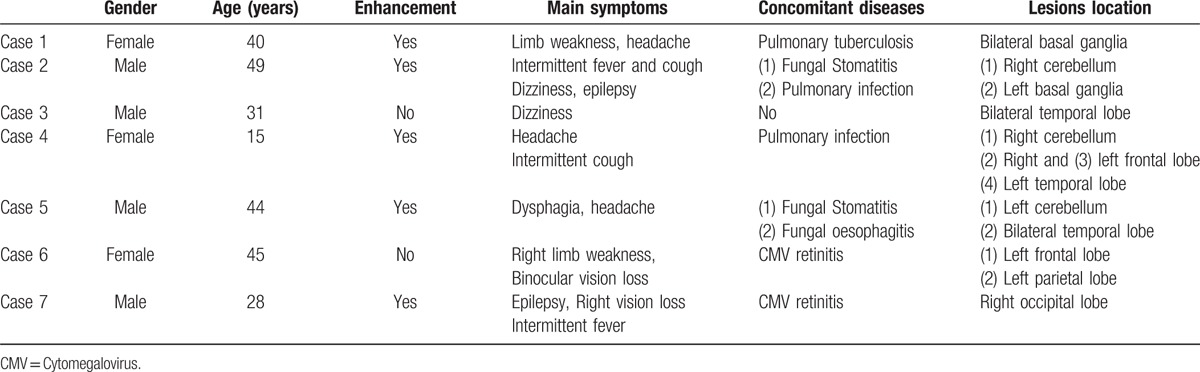
Patient clinical data.

### 2.2 Lesion site and size

**Figure 1:**
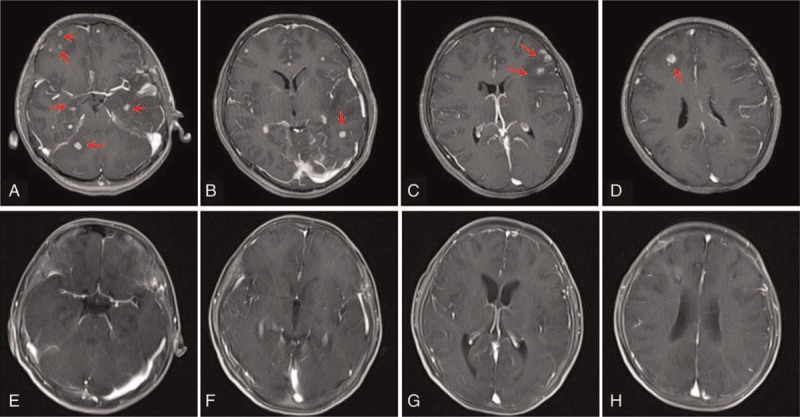
Pre- and postoperative contrast MRI of Case 4: (A–D) preoperation, multiple intracranial lesions (right cerebellum, right and left frontal lobe, left temporal lobe) could be found, as indicated by the red arrows. (E–H) 3 months postoperation, after the suitable treatments, the multiple intracranial lesions disappeared. MRI = magnetic resonance imaging.

The lesions were located in the cerebellum in 3 cases, in the temporal lobe in 3 cases, in the frontal lobe in 2 cases, in the parietal lobe in 1 case, in the occipital lobe in 1 case, and in the basal ganglia in 2 cases, and there were multiple intracranial lesions in 6 patients (Table 1, Fig. 1). The lesion sizes varied from 0.2 cm×0.3 cm×0.5 cm to 2 cm×5 cm×7 cm.

### 2.3 Laboratory tests

The toxoplasma IgM and IgG and cytomegalovirus IgM and IgG in blood serum; cytomegalovirus DNA (deoxyribonucleic acid) and Cryptococcus antigen in cerebrospinal fluid (CSF); and routine CSF were examined (Table 2).

**Table 2:**
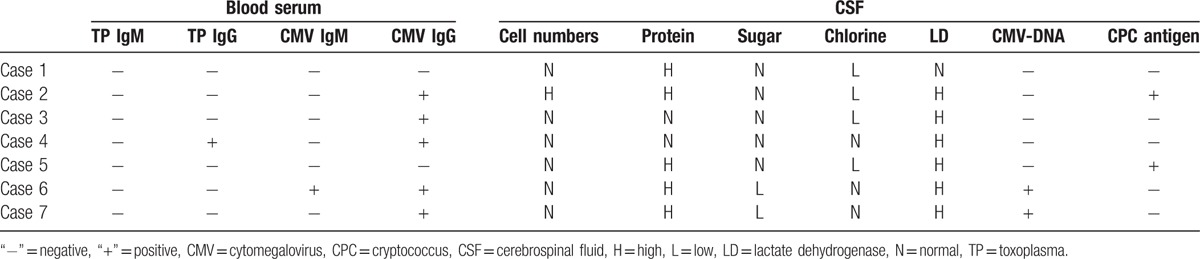
Laboratory test results.

### 2.4 Surgical procedure

A stereotactic frame (Leksell G type, Sweden) was installed in the patients under local anesthesia; 3.0 T MRI scans (layer thickness: 2 mm) was taken, and T1 enhancement (when enhanced nodularity could be found in the lesions) or T2 FLAIR was used to measured and calculate the coordinates of the intracranial lesions.^[10]^ The trajectory was selected according to which vascular and functional areas of the brain could be avoided. Biopsy specimens were collected from 2 different lesions (in 1 case) or from only a single lesion when there were multiple lesions with similar imaging characteristics.

Due to the infectious nature of AIDS, special care was taken to protect the operating room staff. During surgery, the surgeon put on a face mask, protective glasses, disposable surgical gowns, and double gloves. A scalp incision and burr hole were made under local anesthesia. After the dura was opened, an arc was installed, and the coordinates were checked. A side-cut aspirating biopsy needle (with a diameter of 2.5 mm, Leksell, Sweden) was used to obtain tissue specimens from 4 directions. Each specimen was ∼2 × 2 × 10 mm in size and fixed in 10% formalin. The samples were examined with hematoxylin-eosin (HE) staining and periodic acid-Schiff (PAS) staining as well as immunohistochemical study with monoclonal antibodies.

After the operation, the patients remained in the neurosurgical department for overnight observation. The respiration, heart rate, blood pressure, pulse, pupil response, and patient awareness were monitored. Usually prophylactic anti-epilepsy treatment was given. A head CT scan was performed on the day following operation.

## 3 Results

### 3.1 Histological result and clinical diagnosis

**Figure 2:**
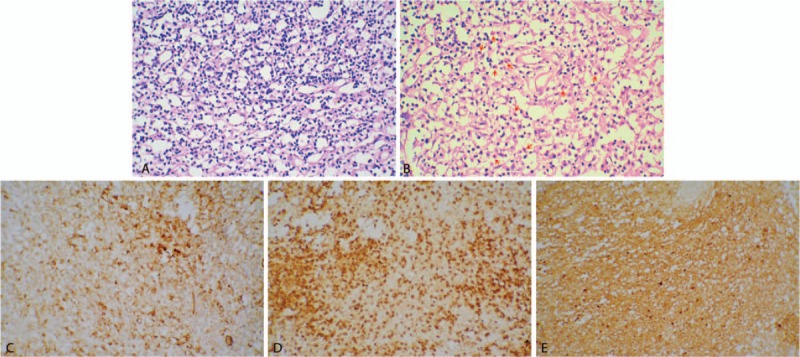
Pathological results of Case 4 (×200): (A) HE staining showed obvious proliferation of microvascular, microglia reactive hyperplasia with infiltration of lymphocytes, local brain tissue degeneration, and necrosis. (B) PAS staining showed small and red dye particles in the foam-like cells, as indicated by the red arrows. Immunohistochemical staining showed: (C) CD31(+), (D) LCA (+), (E) Protein S100 (+). CD31 = platelet endothelial cell adhesion molecule-1, angiogenic marker, HE = Hematoxylin–eosin, LCA = lymphocytotoxic antibody, related to the immune system abnormalities, predominantly lymphopenia, PAS staining = Periodic Acid-Schiff staining, Protein S100 = neurogenic marker.

All patients were successfully sampled, and the histological results included inflammation in 4 cases, toxoplasma gondii infection in 1 case, astrocytoma (WHO II) in 1 case, and abscess in 1 case. Taking the histological results, laboratory tests, imaging findings, and clinical data together, the clinical diagnosis included TE in 2 cases, Cryptococcus encephalitis in 2 cases, CMV encephalitis in 2 case, tubercular abscess in 1 case, and astrocytoma in 1 case (WHO II). Co-infection with Cryptococcus encephalitis was found in 1 patient with TE. In cases 3 and 4, the clinical diagnosis was directly obtained from histological results (Fig. 2). The diagnostic yield was 100% (Table 3).

**Table 3:**
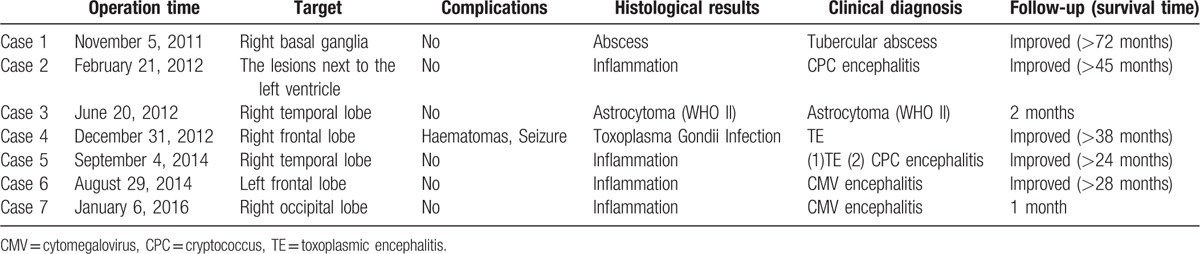
Histological results and clinical diagnosis.

### 3.2 Complications

Intraoperative biopsy-related cerebral hemorrhage in the sampling area was documented in 1 patient, who also had an early postoperative seizure. The overall morbidity was 14.3% (1/7), and there was no operation-related mortality. The hematoma gradually absorbed, without further seizures, under conservative treatment. There were no neurological disorders, intracranial infections, or other serious complications.

### 3.3 Therapeutic results

Based on the clinical diagnosis obtained from the biopsy, the therapies were adjusted in 6 out of 7 patients. Therapies were not adjusted in the patient who was diagnosed with astrocytoma (WHO II), refused further treatment, and died 2 months later. One patient died 1 month later with a diagnosis of Cytomegalovirus encephalitis; his CD4 level was only 1/microliter, and his death was not directly related to the biopsy. Another 5 patients were followed up for 2 to 6 years; 1 patient fully recovered (Fig. 1), and the remaining 4 patients improved significantly. The median survival time was longer than 28 months (range from 24 to 72 months, Table 3).

## 4 Discussion

Although CT and MRI scanners can give us the accurate position of intracranial lesions, they lack the specificity required to secure a firm etiological diagnosis.^[11]^ We searched the published English literature about stereotactic biopsy in AIDS. Databases, including PubMed, EbscoHost, and Ovid, were searched for the keywords “biopsy,” “brain,” “HIV,” “AIDS,” and variations of these words. Only human studies published from 1985 to 2016 were considered. Studies were included if they reported original research data on the diagnostic rate of brain stereotactic biopsy in HIV/AIDS patients and the final histopathological diagnosis from brain stereotactic biopsy. Studies were excluded if they met any of the following criteria: a patient population <10; a study population already included in another study; a patient cohort retrospectively selected based on a certain diagnosis, such as PML; the samples were collected by another form of biopsy, such as craniotomy; incomplete data; and the manuscript was a commentary, technical note or review. A total of 19 studies were included for review^[3,5,10,12–27]^ (Table 4). The average positive biopsy rate, complication rate, and hemorrhage morbidity and mortality were 80.00% to 100% (mean 90.98%, 746/820), 0% to 20.00% (mean 5.93%, 37/624), 0% to 18.18% (mean 3.84%, 24/624), and 0% to 9.09% (mean 2.08%, 13/624), respectively.

**Table 4:**
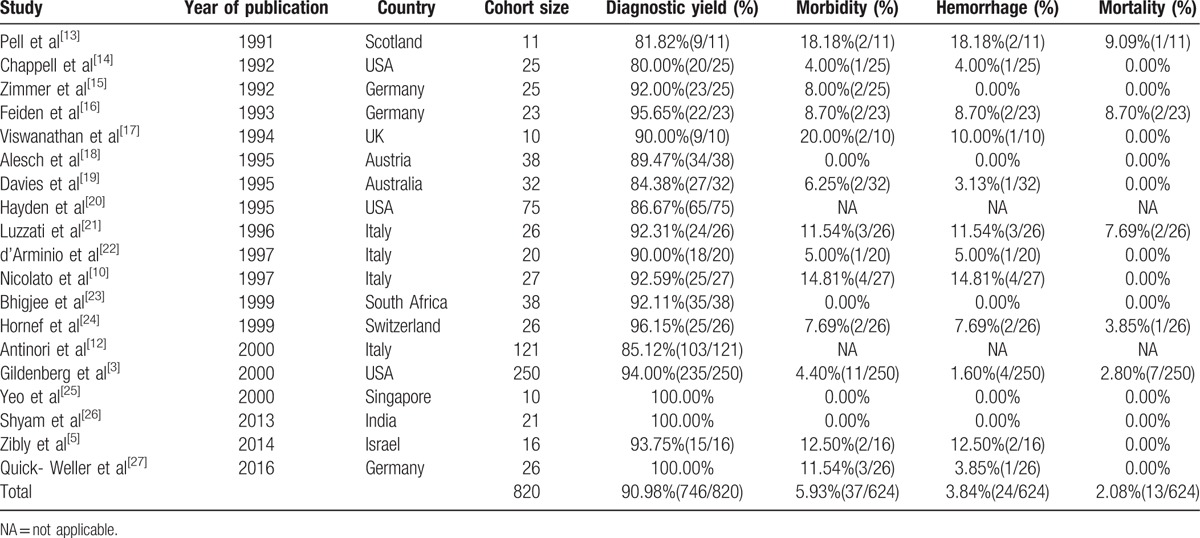
Literature of stereotactic biopsy in AIDS patients (1).

The spectrum of neurologic disease that complicates HIV infection is extremely broad. Similarly, we also analyzed the diagnostic results in the literature and found the following: in 17 studies^[3,5,10,13–25,27]^ with 678 patients, there were 208 cases of lymphoma (30.68%), 171 cases of PML (25.22%), 131 cases of TE (19.32%), 48 cases of HIV encephalitis (7.08%), 5 cases of CMV (0.74%), 26 cases with multiple diagnoses (3.83%), and 48 cases with nonspecific positive results (7.08%) (Table 5). Obviously, the most common intracranial lesions included lymphoma, PML, and TE. However, some other papers showed a different pattern.^[5,13,15,17,18,22,23]^ We had a similar result in our study; the clinical diagnosis included TE in 2 cases (28.6%), Cryptococcus encephalitis in 2 cases (28.6%), CMV encephalitis in 2 cases (28.6%), astrocytoma in 1 case (14.3%), and tubercular abscess in 1 case (14.3%); no lymphoma cases were observed.

**Table 5:**
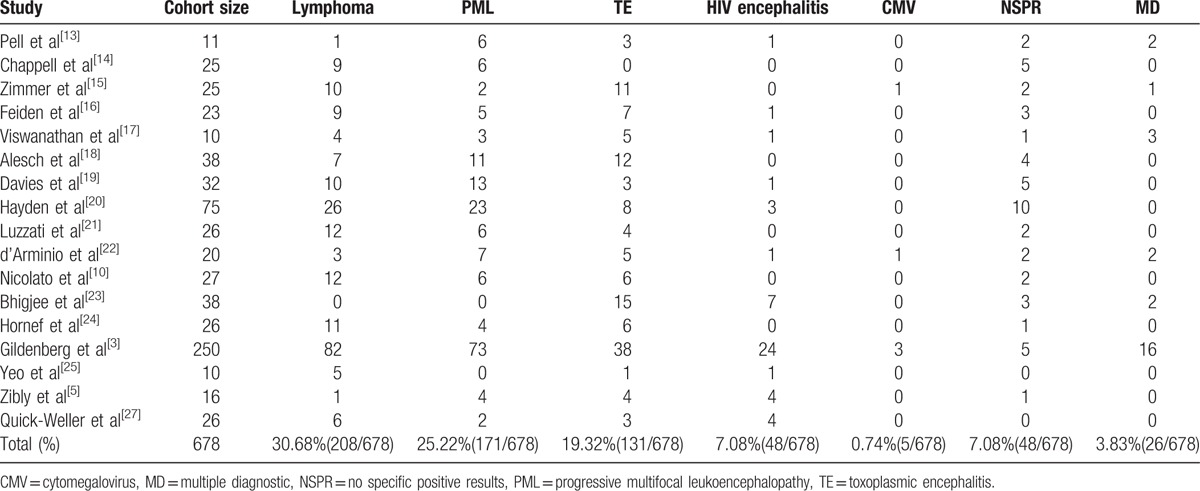
Literature of stereotactic biopsy in AIDS patients (2).

To explain the differences in the proportions of HIV-related focal brain lesion-causing disorders, several possibilities are considered. Ammassari^[28]^ compared the years following the introduction of highly active antiretroviral therapy (HAART) with the pre-HAART era for trends in the proportions of HIV-related focal brain lesion-causing disorders. They found that the major diagnoses in the 281 patients were toxoplasmic encephalitis (36.4%), primary CNS lymphoma (26.7%), progressive multifocal leukoencephalopathy (18.2%), and focal HIV encephalopathy (5.0%). They concluded that since the introduction of HAART, the incidence of toxoplasmic encephalitis has decreased or stabilized, whereas CNS lymphoma has dramatically declined. According to our observation, lymphoma usually occurs in the relatively later period for AIDS patients. It is reasonable that with early diagnosis and treatment, the lymphoma morbidity in AIDS patients decreased. Otherwise, our study and the other reports^[5,13,15,17,18,22,23]^ enrolled a relatively small number of patients. A larger cohort of patients is needed to establish more definitive conclusions.

To improve the diagnostic yield rates and reduce the morbidity, significant attention has been paid to the procedure, including surgical planning to determine the biopsy trajectory, imaging technique, and target choice. Non-necrotic areas or lesions with marked enhancement components are more suitable, whereas the mostly central part of the lesions is necrotic, and the biopsy positive rate may be low. The common postoperative complications of stereotactic biopsy were hemorrhage, edema, epilepsy, infections, and more, whereas the most frequent complication was hemorrhage.^[10,29]^ In this study, 1 patient had an early sampling area with bleeding and a seizure. To reduce the possibility of post-operation hemorrhage, the preoperative platelet count should be more than 100,000/mL,^[30]^ and careful surgical planning should be performed to avoid the vessels within the trajectory.^[31]^ Postoperative CT scans should be regularly performed to find the hemorrhage as early as possible.

Stereotactic MRI guided biopsy is a minimally invasive procedure with low morbidity and high diagnostic accuracy for diagnosing and grading brain lesions. The diagnostic accuracy of stereotactic biopsy can be further enhanced by the careful interpretation of neuroradiological and clinical information. Our results differ from previous reports in that the study results were divided into histological results and clinical diagnoses. It is easy to discriminate between tumors and infections, but it is usually very hard to histologically discriminate between different infections types contributing to brain lesions in HIV patients. Very few cases have typical histological characteristics for making a definite pathologic diagnosis. In our study, only 2 of 7 cases were clinically diagnosed from direct histological findings. In other cases, serum and CSF laboratory testing, imaging data (single or multiple lesions, with or without edema or enhancement, etc.), and empirical or diagnostic treatment results were incorporated with histological results to make a final, correct clinical diagnosis. If the patient's symptoms, images, and/or biochemical test results improved, we could confirm the correctness of the diagnosis.

## 5 Conclusion

The stereotactic biopsy of cerebral lesions is an extremely safe, effective procedure for evaluating intracranial lesions in AIDS patients, establishing a tissue-based diagnosis of CNS lesions, and developing suitable treatments in AIDS patients, and this approach has an acceptable risk/benefit ratio. However, in some cases, the histological diagnosis should be integrated with other information to make a correct clinical diagnosis. On the other hand, the development and application of molecular pathology to diagnose intracranial AIDS lesions may improve the future diagnostic yield of stereotactic biopsies.^[32,33,34]^

## Acknowledgments

The authors like to thank Jincao Chen and Pucha Jiang for their valuable discussion. The authors thank all participants in the surgery. In addition, they would like to thank the editor and anonymous reviewers, who have helped to improve the paper.
